# Acacetin exerts antioxidant potential against atherosclerosis through Nrf2 pathway in apoE^−/−^ Mice

**DOI:** 10.1111/jcmm.16106

**Published:** 2020-11-26

**Authors:** Yao Wu, Fei Song, Yunda Li, Jingzhou Li, Yukai Cui, Yixiang Hong, Weimin Han, Weiyin Wu, Ishan Lakhani, Gang Li, Yan Wang

**Affiliations:** ^1^ Xiamen Cardiovascular Hospital, Xiamen University Xiamen China; ^2^ Laboratory of Cardiovascular Physiology Li Ka Shing Institute of Health Sciences Hong Kong China

**Keywords:** acacetin, apoE^−/−^ mice, atherosclerosis, endothelial cell, oxidative stress

## Abstract

Oxidative stress has a considerable influence on endothelial cell dysfunction and atherosclerosis. Acacetin, an anti‐inflammatory and antiarrhythmic, is frequently used in the treatment of myocarditis, albeit its role in managing atherosclerosis is currently unclear. Thus, we evaluated the regulatory effects of acacetin in maintaining endothelial cell function and further investigated whether the flavonoid could attenuate atherosclerosis in apolipoprotein E deficiency (apoE^−/−^) mice. Different concentrations of acacetin were tested on EA.hy926 cells, either induced or non‐induced by human oxidized low‐density lipoprotein (oxLDL), to clarify its influence on cell viability, cellular reactive oxidative stress (ROS) level, apoptotic ratios and other regulatory effects. In vivo, apoE^−/−^ mice were fed either a Western diet or a chow diet. Acacetin pro‐drug (15 mg/kg) was injected subcutaneously two times a day for 12 weeks. The effects of acacetin on the atherosclerotic process, plasma inflammatory factors and lipid metabolism were also investigated. Acacetin significantly increased EA.hy926 cell viability by reducing the ratios of apoptotic and necrotic cells at 3 μmol/L. Moreover, 3 μmol/L acacetin clearly decreased ROS levels and enhanced reductase protein expression through MsrA and Nrf2 pathway through phosphorylation of Nrf2 and degradation of Keap1. In vivo, acacetin treatment remarkably attenuated atherosclerosis by increasing reductase levels in circulation and aortic roots, decreasing plasma inflammatory factor levels as well as accelerating lipid metabolism in Western diet‐fed apoE^−/−^ mice. Our findings demonstrate the anti‐oxidative and anti‐atherosclerotic effects of acacetin, in turn suggesting its potential therapeutic value in atherosclerotic‐related cardiovascular diseases (CVD).

## 1 INTRODUCTION

Atherosclerosis‐related cardiovascular disease (CVD) is a leading cause of mortality in developed and developing countries. Atherosclerosis is a chronic inflammatory condition characterized by dyslipidaemia and oxidative stress.^1^ Low‐density lipoprotein (LDL) is considered to be a key molecular in every stage of atherosclerosis. Specifically, it is the oxidized LDL (oxLDL) particles, as opposed to normal LDL, that have a pathogenic influence, activating the vascular intima and subsequently initiating atherosclerosis. In addition to their lipid‐lowering effects in clinical therapies, statins, according to many studies, possess anti‐oxidative effects^2^; as such, given the central role of statins in management, anti‐oxidation is a topic of considerable interest in atherosclerosis research.

Reactive oxygen species (ROS), the major causes of oxidative stress, are generated as metabolic byproducts, and typically include the superoxide anion (•O^2−^), hydroxyl radical (•OH), as well as hydrogen peroxide (H_2_O_2_).^3^ Superoxide dismutase (SOD) disproportionately converts unstable free radical •O^2−^ into H_2_O_2_ and singlet oxygen (^1^O2).^4^ H_2_O_2_ is then resolved into H_2_O and O_2_ by catalase (CAT), glutathione peroxidase (GSH‐PX), and thioredoxin peroxidase (Trx‐PX). Alternatively, H_2_O_2_ is also prone to use chloride ions to generate the more potent oxidizing agent, hypochlorite (HClO),^5^ which may then react with •O^2−^ to produce •OH, thereby amplifying the oxidative stress load on organisms.^6^ Therefore, the dynamic equilibrium state of oxidation and anti‐oxidation is of considerable importance to cell viability, particularly in intimal or endothelial cells. In the development of atherosclerosis, an oxLDL‐mediated activation of the endothelium enhances P‐selectin expression, leading to the recruitment of monocytes from circulation followed by their resultant adhesion to the intimal surface. This immune cascade reaction causes large amounts of inflammatory factors (E‐selectin, VCAM‐1, ICAM‐1, CCL2, CCL5, etc) to be released, strengthening monocyte adhesion, rotation, along with subsequent infiltration into the intima and chemotaxis to macrophages.^7^, ^8^ As a result, the maintenance of the redox equilibrium in endothelial cells as well as the accompanying vascular barrier is both important tasks necessary to facilitate the prevention and treatment of atherosclerosis.

Acacetin, a natural flavone widely distributed in plant pigments, has been shown by many studies to have multiple beneficial biological effects in cancers,^9^, ^10^ cardiac remodelling,^11^ microbial infections,^12^ inflammation ^13^ and oxidative stress.^14^ In human umbilical vein endothelial (HUVEC) cells, acacetin inhibited E‐selectin expression through the p38/MAPK pathway and activation of the nuclear factor NF‐κB.^15^ Acacetin has also demonstrated an ability to down‐regulate inflammatory iNOS and COX‐2 gene expression in RAW264.7 cells by inhibiting the activation of NF‐κB through interfering with the PI3K/Akt/IKK and MAPK pathways.^16^ Moreover, our previous study found that AMPK‐mediated Nrf2 activation through acacetin is involved in cardiomyocyte protection against hypoxia/reoxygenation injury by its anti‐oxidative, anti‐inflammatory and anti‐apoptotic effects.^17^ Collectively, this evidence clearly illustrates the involvement of acacetin in oxidative stress and inflammation‐associated conditions.

Methionine, a thiol amino acid, is not only an initiation amino acid but also a sensitive target for oxidants, giving it vital roles in some critical signalling pathways. Met is easily oxidized to MetO, which is reduced back to Met exclusively by the intracellular methionine sulphoxide reductase (Msr) system. Specifically, MsrA, an enzyme involved in this system, reduces methionine‐*S*‐sulfoxide (MetSO) to Met.^18^ We previously demonstrated that exogenous reconstructed MsrA protein plays a protective role against oxidative stress as well as inflammation in RAW264.7 cells, and attenuates the atherosclerotic process in western diet‐fed apoE deficiency (apoE^−/−^) mice.^19^ However, it is currently still unclear precisely how MsrA exerts protection against oxidative stress.

The goal of this study is to investigate whether acacetin can protect against oxidative stress in humans and attenuate atherosclerosis in apoE^−/−^ mice. Moreover, we will attempt to reveal the mechanisms underlying the role of acacetin in the MsrA‐ and Nrf2‐related pathways, aiming to provide evidence of its potential therapeutic role in atherosclerosis‐related CVD.

## 2 MATERIALS AND METHODS

### 2.1 Reagents

Acacetin (5,7‐dihydroxy‐40‐methoxy flavone) was synthesized in the laboratory as described previously in the United States Patent. Human high oxidi ed low‐density lipoprotein (oxLDL) was obtained from Yiyuan Biotechnologies. Dulbecco's modified Eagle's medium (DMEM), Opti‐MEM I, foetal bovine serum (FBS), antibiotics (100 mg/mL streptomycin and 100 U/mL penicillin), 0.25% trypsin, 2'7'‐dichlorofluorescein diacetate (DCFH‐DA) and lipofectamine^TM^ RNAiMAX transfection reagent were purchased from Thermo Fisher Scientific. Annexin V‐fluorescein (Annexin V‐FITC)/PI apoptosis detection kit was purchased from Dojindo Molecular Technologies. MsrA, NF‐E2‐related factor 2 (Nrf2), kelch‐like ECH‐associated protein 1 (Keap1), sirtuin (SIRT1) and scrambled small interfering RNAs (siRNAs) were purchased from Santa Cruz Biotechnology. TGX^TM^ and TGX Stain‐Free^TM^ FastCast^TM^ Acrylamide kits were purchased from Bio‐Rad. The anti‐Bcl2, anti‐Bax, anti‐Nrf2, anti‐phospho S40 Nrf2 (pNrf2^S40^), anti‐MsrA, anti‐Keap1, anti‐haeme oxygenase‐1 (HO‐1), anti‐Thioredoxin (Trx), anti‐ SIRT1, anti‐catalase (CAT), anti‐SOD1, anti‐ATP‐binding cassette A1 (ABCA1), anti‐ATP‐binding cassette G1 (ABCG1), anti‐GAPDH, anti‐iNOS, anti‐CD206 first antibodies as well as donkey anti‐rabbit (Alexa Fluor® 488) and donkey anti‐mouse (Alexa Fluor® 647) secondary antibodies were all purchased from Abcam. Anti‐Keap1, anti‐SOD2, anti‐AMPKα 1/2, anti‐phospho T172 AMPKα (pAMPK^T172^) and anti‐β‐actin antibodies were purchased from Santa Cruz Biotechnology. The anti‐Caspase3 antibody was obtained from cell signalling technology. Anti‐scavenger receptor class B type I (SR‐BI) and anti‐serum amyloid A (SAA) antibodies were obtained from NOVUS biologicals. Horseradish peroxidase (HRP)‐conjugated anti‐rabbit or mouse secondary antibodies were purchased from Jackson ImmunoReaserch. Mouse enzyme‐linked immunosorbent assay (ELISA) kits: interleukin‐6 (IL‐6), interleukin‐10 (IL‐10), tumour necrosis factor‐alpha (TNFα), monocyte chemotactic protein 1 (MCP‐1), interleukin‐1 alpha (IL‐1α), interleukin‐1 beta (IL‐1β) and granulocyte‐macrophage colony‐stimulating factor (GM‐CSF) were purchased from eBioscience.

### 2.2 Cell culture

Human endothelial cell line EA.hy926 cells were obtained from ATCC. The cells were cultured in DMEM with 10% FBS and 1% antibiotics at 37°C. Cells were seeded in 10 cm, 6‐ or 12‐ well plates and incubated with 2% FBS DMEM at 37°C overnight before further treatment.

### 2.3 Flow cytometry

Flow cytometry was used to determine intracellular ROS levels and the apoptotic status of cells. Intracellular ROS levels were detected by the sensitive fluorescent DCFH‐DA. EA.hy926 cells were pre‐incubated with or without acacetin (0.3 μmol/L, 1 μmol/L, 3 μmol/L) for 24 hours, and then incubated with 20 μmol/L DCFH‐DA for 30 minutes. After washing with phosphate buffer (PBS), the cells were exposed to 50 μg/mL high oxLDL for 15 minutes. The DCF fluorescence intensity was measured by flow cytometry (Beckman Coulter).

For the apoptotic status study, cells were pretreated with or without acacetin at different concentrations (0.3 μmol/L, 1 μmol/L, 3 μmol/L) for 4 hours and then incubated with 5 μg/mL high oxLDL for another 20 hours. After gently washing with precooled PBS, cells were collected and resuspended in binding buffer within Annexin V‐FITC dye for 30 minutes followed by PI staining for 5 minutes at room temperature.

### 2.4 siRNA transfection

EA.hy926 cells were seeded in 6‐ or 12‐well plates. The cells were transfected with control siRNA, MsrA siRNA, Nrf2 siRNA, Keap1 siRNA or SIRT1 siRNA (50 nmol/L) in Opti‐MEM I using lipofectamine^TM^ RNAiMAX. After 12 hours, the medium was changed to DMEM with 10% or 2% FBS and then treated with or without acacetin or high oxLDL for further study.

### 2.5 Western blot analysis

Harvested cells or frozen tissues were lysed by RIPA with 1% protease and phosphatase inhibitors (Roche) for Western blot assay. The appropriate amount of proteins was loaded and separated by 10% or 12% SDS‐PAGE and transformed onto PVDF membrane. Protein expression was detected by primary antibodies followed by HRP‐conjugated secondary antibodies. Signals were detected using an enhanced chemiluminescence kit (ECL, GE Healthcare) and captured by a chemiluminescence detection system (FlouChem E). The band densitometry was analysed by Image J software (NIH).

### 2.6 Animals

ApoE^−/−^ mice on the C57BL/6 background were purchased from Vital River Laboratory Animal Technology Company and housed in micro isolator cages at the Xiamen University Laboratory Animal Center. Mice were fed in a temperature‐controlled facility (temperature 22 ± 1°C, humidity 60%, 12‐12h dark‐light cycle) with free access to food and water. Animal care and experimental procedures were performed under the regulations of the Institutional Animal Care and the Ethics Committee for animal experiments at Xiamen University, in accordance with the guidelines for the Care and Use of Laboratory Animals of the Chinese Welfare Committee. Thirty female apoE^−/−^ mice at 21 weeks of age were randomly and equally divided into three groups and subcutaneously injected with acacetin (dose of 15 mg/kg) or normal saline or shame, respectively. Mice were fed an AIN76A Western diet for twelve weeks to accelerate the development of atherosclerosis.

### 2.7 Determination of basic biochemical parameters in Western diet‐fed mice

After 12 weeks of experimental procedures, blood samples were collected from mice after overnight fasting by retro‐orbital venous plexus puncture. Plasma was immediately separated by centrifugation at 1000× *g* for 10 minutes at 4°C. Total cholesterol (TC), triglyceride (TG), high‐density lipoprotein cholesterol (HDL‐C), low‐density lipoprotein cholesterol (LDL‐C) and apolipoprotein AI (apoAI) levels were measured by enzymatic colorimetric methods using Mind Bioengineering kits. The remaining plasma was used for determination of IL‐6, IL‐10, TNFα and MCP‐1 levels by ELISA kits according to the manufacturer's instructions. Frozen mouse livers were lysed by RIPA with 1% proteinase inhibitors for Western blot.

### 2.8 Histochemical and immunohistochemistry of atherosclerotic lesions

Mice were subcutaneously injected with acacetin or normal saline two times every day for 12 weeks. Mice were made to fast after the last injection and then sacrificed. The aortic roots were embedded in OCT (Sakura, USA) and quickly frozen horizontally to −20℃. Eight micrometers serial sections of the aortic root were collected on 10 slides.

For atherosclerosis analysis, the entirety of the aorta was fixed in 4% paraformaldehyde, opened longitudinally, and then analysed *en face*. The aortic root slides were determined by Oil Red O (ORO) staining and quantification by Image J software as described previously.^19^

For plaque component analysis, immunohistochemistry was carried out using Abcam's IHC staining protocol for frozen sections. Briefly, the aortic root slides were fixed in precooled acetone and then stained with anti‐iNOS, anti‐CD206, anti‐pNrf2^S40^, anti‐Nrf2 and anti‐MsrA primary antibodies followed by donkey anti‐rabbit (Alexa Fluor® 488) or anti‐mouse (Alexa Fluor® 647) secondary antibodies, respectively. Images were captured using the Leica SP8 fluorescent microscope.

### 2.9 Statistical analysis

Data are presented as mean ± SEM. Statistical analyses were performed using Onaway ANOVA between groups. Differences were considered to be significant at *P* < .05.

## 3 RESULTS

### 3.1 Acacetin inhibited high oxLDL‐induced cell death and oxidation

**Figure 1:**
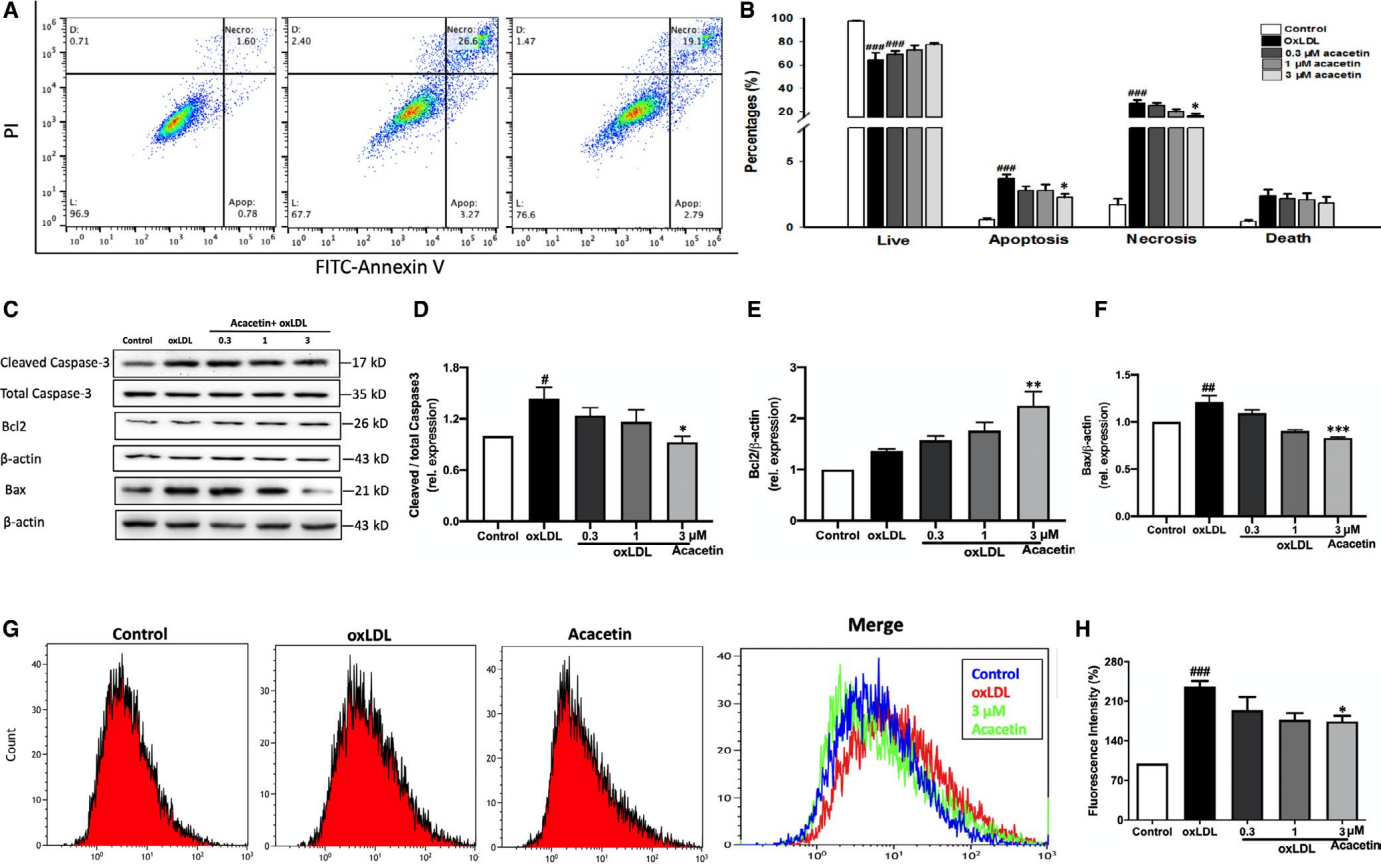
Effects of acacetin on cell apoptosis and intracellular ROS in EA.hy926 cells under oxLDL stimulation. (A‐B) EA.hy926 cells were pretreated with different concentrations (0.3, 1, 3 μmol/L) or without acacetin and stimulated with 5 μg/mL oxLDL. Apoptosis was quantified by flow cytometry. Live (L); Apoptosis (Apop); Necrosis (Necro); Death (D). (C‐F) Cellular cleaved caspase‐3, total caspase‐3, Bcl2 and Bax protein expression levels were determined by Western blots. (G‐H) Intracellular ROS levels were measured by flow cytometry. EA.hy926 cells were pretreated with different concentrations (0.3, 1, 3 μmol/L) or without acacetin for 24 h followed by 50 μg/mL oxLDL for 15 min. n = 5 for each group, *P* ^#^ < .05, *P* ^##^ < .01, *P* ^###^ < .001 vs control group; *P* ^*^ < .05, *P* ^**^ < .01, *P* ^***^ < .001 vs oxLDL‐stimulated group

MTT method was used to determine the optimal concentration of high oxLDL for the following studies. The cytotoxicity of high oxLDL was tested on EA.hy926 cells, revealing that high oxLDL, unlike normal oxLDL, had stronger cytotoxicity that induced much more cell death after 24 hours incubation (Figure S1A). We chose 5 μg/mL high oxLDL, which caused nearly 28% cell death, as the final study concentration. Pretreatment with acacetin for 4 hours significantly protected against the oxLDL‐mediated reduction of cell viability (Figure S1B). To determine the deep effects of acacetin on cell death, cells were treated with the same procedures as in the MTT study. Compared to high oxLDL‐treated cells, cells treated with acacetin showed a remarkable reduction in apoptosis (2.23% ± 0.31% vs 3.69% ± 0.31%) and necrosis (16.10% ± 1.97% vs 26.52% ± 3.53%) (Figure 1A,B) through reduced cleaved caspase‐3 activation, Bax protein expression as well as increased Bcl2 protein expression (Figure 1C‐F).

In addition to this, we also measured the intracellular ROS levels. Cells were pretreated with acacetin for 24 hours followed by 20 μmol/L DCFH‐DA and 50 μg/mL high oxLDL for 15 minutes. As shown in Figure 1G,H, high oxLDL treatment resulted in a high intracellular ROS level (the fluorescence intensity nearly 2.36 folds of the control group). In contrast, ROS production was markedly reduced in cells pretreated with 3μM acacetin (decreased to 1.4 folds of control). Moreover, the oxidase protein and MDA were also reduced by different concentrations of acacetin (Figure S1C,D). These results in turn indicate that acacetin could protect EA.hy926 cells from high oxLDL‐induced cell death by reducing intracellular ROS levels.

### 3.2 Acacetin enhanced cellular anti‐oxidative defence through increasing oxidoreductases expression

To investigate how acacetin exerts its anti‐oxidative stress effects, EA.hy926 cells were treated with acacetin at different concentrations for 24 hours. Three micrometers acacetin significantly increased reductase MsrA, Nrf2, Nrf2 downstream HO‐1, and CAT protein expression at the cellular basal level (Figure S1E‐S1I). These results suggest that acacetin may enhance basal anti‐oxidative defences.

**Figure 2:**
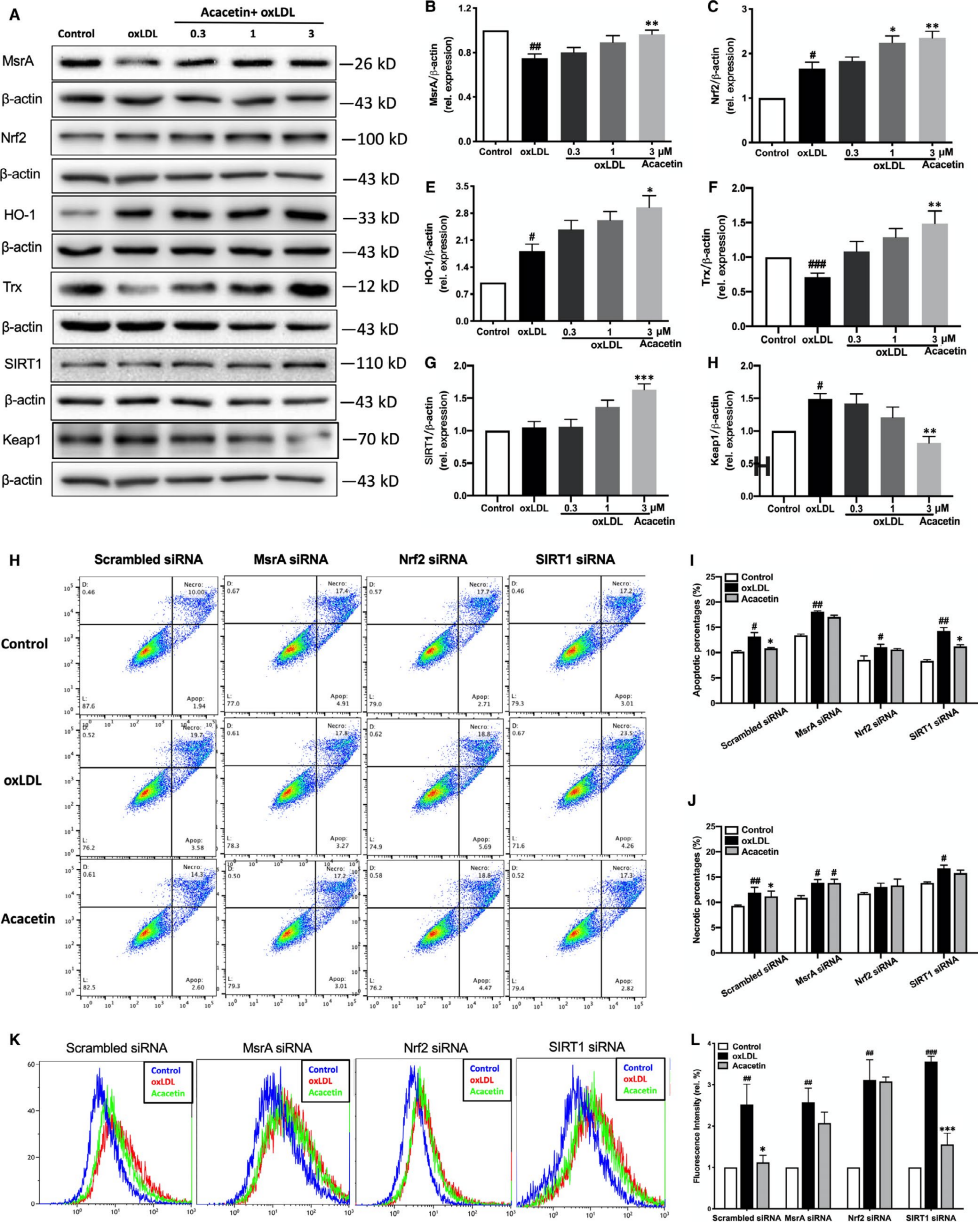
Regulatory effects of acacetin on anti‐oxidative stress‐related reductases as well as the abolishment of its protective effects against oxLDL in EA.hy926 cells with the silencing of MsrA or Nrf2. (A‐G) EA.hy926 cell MsrA, Nrf2/Keap1‐related protein, and SIRT1 expression levels without (control) or with oxLDL stimulation in the absence (oxLDL) or presence of 0.3, 1, or 3 μmol/L acacetin were measured by Western blot. (H‐J) Apoptosis of EA.hy926 cells was measured by flow cytometry. Cells were transfected with scrambled siRNA, MsrA siRNA, Nrf2 siRNA or SIRT1 siRNA for 48 h, and then subjected to oxLDL treatment in the absence (control) or presence of 3 μmol/L acacetin. (K‐L) Intracellular ROS levels were measured by flow cytometry. Cells were transfected with scrambled siRNA, MsrA siRNA or Nrf2 siRNA for 48 h, and then subjected to oxLDL treatment for 15 min in the absence (control) or presence of 3 μmol/L acacetin for 24 h. n = 5 for each group, *P* ^#^ < .05, *P* ^##^ < .01 vs control group; *P* ^*^ < .05, *P* ^**^ < .01, *P* ^***^ < .001 vs oxLDL‐stimulated group

Furthermore, in order to ensure the anti‐oxidative effects of acacetin under oxidative stress conditions, cells were pretreated with acacetin at different concentrations for 4 hours followed by 5 μg/mL high oxLDL stimulation for 20 hours. Surprisingly, we found that MsrA protein expression level was also significantly increased at 3 μmol/L acacetin (Figure 2A,B). Moreover, acacetin also increased Nrf2, downstream HO‐1, Trx (Figure 2C‐E) and SIRT1 protein expression levels (Figure 2F), but did not change pAMPK^Thr172^/ tAMPK levels (Figure S1N). Interestingly, we also showed that 3 μmol/L acacetin remarkably decreased oxLDL‐induced Keap1 expression level (Figure 2G). The Nrf2/Keap1 system is a defence mechanism used to preserve cellular homeostasis, and Nrf2 is regarded as a master regulator of the oxidative stress response.^20^ As such, these results preliminarily confirmed that acacetin can exert anti‐oxidative effects through the Nrf2 pathway but not the AMPK pathway. Acacetin also slightly up‐regulated CAT, SOD1 and SOD2 levels (Figure S1J‐S1M). Collectively, these data suggest that acacetin can potentiate cellular anti‐oxidative defence through the up‐regulation of MsrA, Nrf2, HO‐1, Trx and SIRT1 to reduce intracellular ROS levels, which in turn indicates that acacetin may be involved in Nrf2/Keap1 or MsrA‐related pathways.

### 3.3 Acacetin enhanced cellular anti‐oxidative effects through the MsrA‐Nrf2/Keap1 pathway

In order to confirm whether acacetin exerts its anti‐oxidative stress effects through Nrf2/Keap1 or some other pathway, we silenced cellular *nrf2*, *msra*, *sirt1* gene expression using siRNAs. We displayed that the influence of acacetin on not only easing apoptosis and necrosis (Figure 2H‐J), but also reducing intracellular ROS levels disappeared when Nrf2 or MsrA expression was silenced (Figure 2K‐L); however, it should be noted that acacetin still promoted a clear anti‐apoptotic and mild anti‐necrotic effect when cellular SIRT1 was silenced.

**Figure 3:**
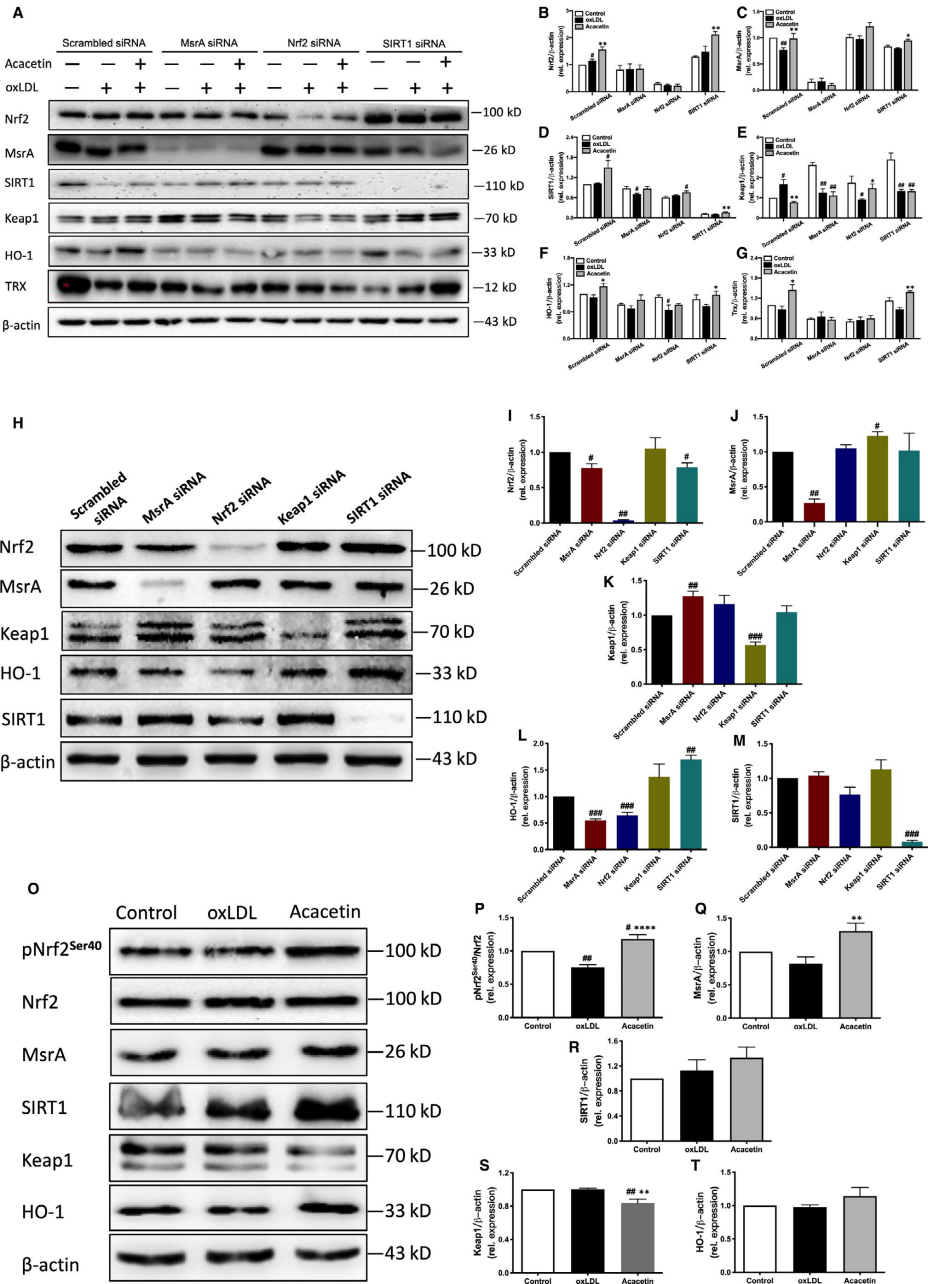
Acacetin exerted anti‐oxidative effects by the MsrA‐Nrf2/Keap1 pathway through the phosphorylation of Nrf2 and inhibition of Keap1 expression. (A‐G) MsrA, Nrf2/Keap1‐related protein, and SIRT1 expression levels without (control) or with oxLDL stimulation in the absence (oxLDL) or presence of 3 μmol/L acacetin after the silencing of MsrA, Nrf2 or SIRT1 protein expression were measured by Western blot. (H‐M) MsrA, Nrf2/Keap1‐related protein and SIRT1 expression levels were analysed by Western blot under MsrA, Nrf2, Keap1 or SIRT1 silencing conditions. (N‐S) Cellular pNrf2^Ser40^, Nrf2, MsrA, SIRT1, Keap1 and HO‐1 expression levels without (control) or with oxLDL stimulation in the absence (oxLDL) or 3 μmol/L acacetin for 2 h were measured by Western blot. n = 5 for each group, *P* ^#^ < .05, *P* ^##^ < .01, *P* ^###^ < .01 vs control group; *P* ^*^ < .05, *P* ^**^ < .01, *P* ^***^ < .001, *P* ^****^ < .0001 vs oxLDL‐stimulated group

We then focused on the changes in Nrf2/Keap1‐ and MsrA‐related oxidoreductases post‐Nrf2, MsrA and SIRT1 gene silencing. Upon Nrf2 silencing, the protective downstream reductases HO‐1 and Trx showed no changes (Figure 3A,F‐G) when pretreated with acacetin followed by 5 μg/mL high oxLDL stimulation. Whilst MsrA expression level was only slightly up‐regulated by acacetin (Figure 3C), the SIRT1 level was significantly increased after acacetin treatment (Figure 3D) which had the same tendency as the scrambled group. Interestingly, Keap1 expression in the scrambled group was remarkably contrary to that when Nrf2 expression was reduced (Figure 3E). Moreover, when MsrA was silenced, aside from the lack of MsrA increase after either oxLDL stimulation or pretreatment with acacetin followed by oxLDL (Figure 3C), the levels of neither Nrf2 nor Nrf2 pathway‐related downstream reductases, HO‐1 and Trx, changed (Figure 3A,F‐G); however, it was found that Keap1 expression was in fact notably decreased with either oxLDL stimulation alone or when it was accompanied by acacetin pretreatment (Figure 3E). The expression of Nrf2, MsrA, HO‐1 and Trx had no changes between the SIRT1 silenced and scrambled groups (Figure 3B‐D,F‐G), albeit Keap1 expression had the same tendency with MsrA silenced group (Figure 3E). These results in turn suggest that the Nrf2 pathway may be related to MsrA.

In order to directly confirm the relationship between MsrA and the Nrf2/Keap1 pathway, we silenced cellular *msra*, *nrf2*, *keap1*, *sirt1* gene expression separately. We found an interesting phenomenon that when the MsrA protein was knocked down by *msra* siRNA (Figure 3J), expression levels of Nrf2 and downstream HO‐1 significantly decreased (Figure 3I,L), Keap1 increased (Figure 3K), and SIRT1 did not change (Figure 3M). On the contrary, MsrA protein expression level showed little difference when Nrf2 was knocked down by *nrf2* siRNA (Figure 3J), whilst SIRT1 demonstrated only a minor reduction (Figure 3M). More noteworthy though was the fact that MsrA expression level was up‐regulated when Keap1 was silenced by *keap1* siRNA (Figure 3J). Our results not only indicate an apparent lack of relation between SIRT1 and MsrA‐related or Nrf2/Keap1 pathways, but also the involvement of Nrf2/Keap1 in MsrA‐related pathways, in turn suggesting that acacetin may in fact exert its anti‐oxidative influence through a MsrA‐Nrf2/Keap1 pathway.

Activation of Nrf2 is mainly dependent on the disruption or inactivation of Keap1 along with its subsequent phosphorylation.^21^ To further study this phenomenon, we investigated the stimulation of Nrf2 by acacetin. After EA.hy926 cells were treated with 3μM acacetin for 2h, phospho‐Nrf2^Ser40^ and downstream HO‐1 expression levels were significantly increased (Figure 3O,S), whereas Keap1 levels were markedly decreased (Figure 3N,R). Further, MsrA protein expression levels were significantly up‐regulated (Figure 3P), with those of SIRT1 only slightly increased (Figure 3Q). These data illustrate that acacetin exerts its anti‐oxidative effects through the MsrA‐Nrf2/Keap1 pathway through both the phosphorylation of Nrf2 at Ser40 and the inhibition of Keap1 expression.

### 3.4 Acacetin attenuated atherosclerosis in Western diet‐fed apoE^‐/‐^ mice through the activation of Nrf2 and MsrA in the lesions

ApoE^−/−^ mice were subcutaneously injected with normal saline or acacetin and fed a Chow (blank group) or Western diet for 12 weeks. The body weights (at various time‐points) of mice showed no difference between the control and acacetin‐treated groups, but spleen/body weight ratios (at the endpoint) were significantly lower in acacetin‐treated mice (Table 1). After 12 weeks, plasma TC, TG, HDL‐C and LDL‐C levels were measured. TC and LDL‐C levels were not different between control and acacetin‐treated groups, albeit TG levels were significantly higher in the latter. Interestingly, HDL‐C and apoAI levels were markedly increased in acacetin‐treated mice (Table 1).

**Table 1:** Basic index, plasma lipid and inflammatory factors in apoE^−/−^ mice

	Blank	Control	Acacetin
Body Weight (g)	24.72 ± 0.40	24.25 ± 1.00	26.92 ± 0.87
Spleen/body ratio	0.50 ± 0.02	1.04 ± 0.17^#^	0.42 ± 0.03**
TC (mmol/L)	12.00 ± 0.58	17.08 ± 1.06^#^	18.59 ± 0.77^#^
TG (mmol/L)	1.38 ± 0.10	1.68 ± 0.11	1.84 ± 0.05
HDL‐C (mmol/L)	13.51 ± 2.41	27.85 ± 7.46	45.93 ± 6.74^#^
LDL‐C (mmol/L)	11.83 ± 0.89	15.02 ± 1.90	14.28 ± 0.98
ApoAI (mg/mL)	0.10 ± 0.01	0.13 ± 0.01	0.18 ± 0.01^*^
IL‐6 (pg/mL)	19.10 ± 2.30	99.40 ± 22.49^#^	41.30 ± 14.01***
TNF‐α (pg/mL)	21.90 ± 3.18	19.70 ± 5.39	16.30 ± 2.58*
IL‐10 (pg/mL)	193.70 ± 14.35	157.60 ± 7.55^#^	227.60 ± 27.97**
MCP‐1 (pg/mL)	415.50 ± 31.54	689.30 ± 153.12	589.40 ± 57.17
IL‐1α (pg/mL)	8.90 ± 1.06	21.80 ± 6.99	17.0 0 ± 4.94
IL‐1β (pg/mL)	21.60 ± 6.27	21.70 ± 6.45	20.50 ± 2.98
GM‐SCF (pg/mL)	107.90 ± 9.20	78.30 ± 4.32^#^	126.90 ± 12.16*

Note

Data are given as the mean ± SEM, n = 10. The statistical analysis was performed using One‐way ANOVA method or Student's *t* test.

Mice were fed a Western‐type diet and injected subcutaneously with normal saline or acacetin for 12 wk.

Abbreviations: TC, total cholesterol; TG, triglycerides.

^#^ Is statistically significant vs blank group.

* Is statistically significant vs control group.

**Figure 4:**
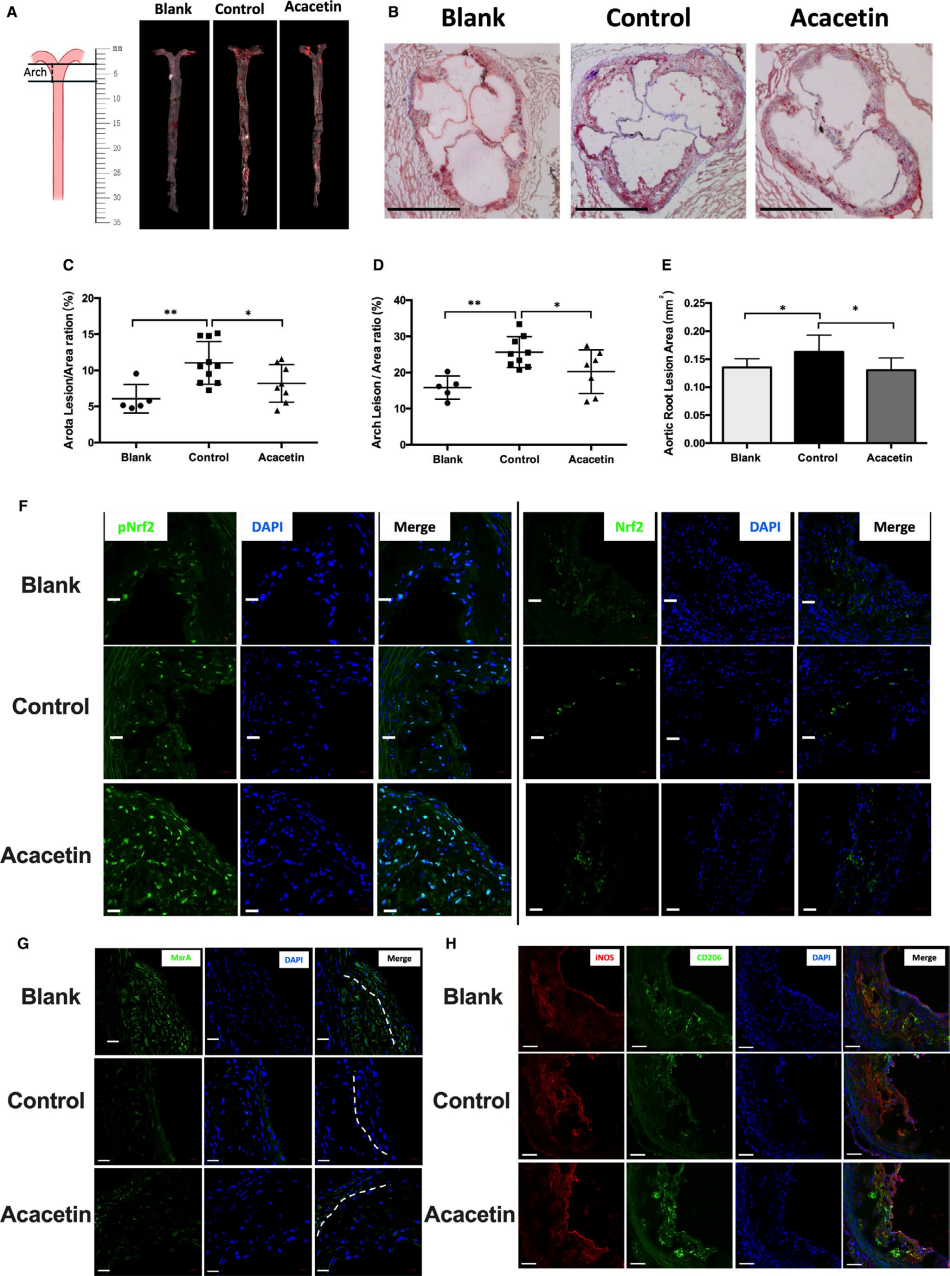
Acacetin attenuated atherosclerosis in Western diet‐fed apoE^−/−^ mice by driving the macrophage phenotype to ‘repair’ M2‐polarized cells through the activation of Nrf2 and MsrA in lesions. (A‐B) The atherosclerotic lesions were examined using Oil Red O‐stained cross‐sections of the aortic root (8‐μm serial sections) and by en face analysis of the aorta. Representative lesions in cross‐sections of the aortic root (n = 5) and en face aortas (n = 5). (C‐E) The atherosclerotic lesion areas were quantified by Image J software. (F) The pNrf2 and Nrf2 expression levels in aortas were detected by immunofluorescence assay. Micrographs were captured at ×400 magnification. (G) The MsrA expression levels in aortas were detected by immunofluorescence assay. Micrographs were captured at ×400 magnification. (H) The M1 (positive with iNos) and M2 (Positive with CD206) macrophages profile in aortas were detected by immunofluorescence assay. Micrographs were captured at ×200 magnification. n = 5‐9 of each group, *P* ^*^ < .05, *P* ^**^ < .01 vs control group

The impact of acacetin injection on the development of atherosclerosis in apoE^‐/‐^ mice was assessed. Representative atherosclerotic lesions in *en face* images and cross‐sections of aortic roots stained with ORO are shown in Figure 4A,B *En face* analysis of pinned‐out aortas revealed that the atherosclerotic lesion percentage area in acacetin‐injected mice (8.19% ± 0.92%) was significantly reduced compared to that of in the control group (11.04% ± 1.04%, *P* < .05, Figure 4D), especially in the arch region (20.25% ± 2.28% vs 25.64% ± 1.42%, *P* < .05, Figure 4C). In addition to this, the lipid staining area in the aortic root lesion of acacetin‐treated mice (0.13 ± 0.01 mm^2^) was 26.7% (*P* < .05) smaller than that of in control mice (0.18 ± 0.02 mm^2^, Figure 4E).

We uncovered a higher expression level of pNrf2^S40^ in both the arterial wall and plaque but Nrf2 mainly in the plaque of acacetin‐injected mice compared to those in control groups, indicating that acacetin could successfully activate Nrf2 in aortic cells (Figure 4F). We also found that MsrA expression recovered mainly in the arterial wall of acacetin‐treated mice (Figure 4G). Moreover, at the endpoint, we stained macrophages to distinguish the phenotype of the different lesions. Interestingly, many CD206 positive ‘repair’ M2‐polarized macrophages, accompanied by only few iNOS positive pro‐inflammatory M1‐polarized macrophages, accumulated in the intima of acacetin‐treated mice, whereas the opposite was observed in the control group (Figure 4H). These data showed that acacetin attenuates the development of atherosclerosis in Western diet‐fed apoE^‐/‐^ mice by driving a pro‐repair M2‐polarized macrophage phenotype through the activation of Nrf2 and MsrA in the lesions.

### 3.5 Acacetin ameliorated oxidative stress and inflammatory in apoE^−/−^ mice

**Figure 5:**
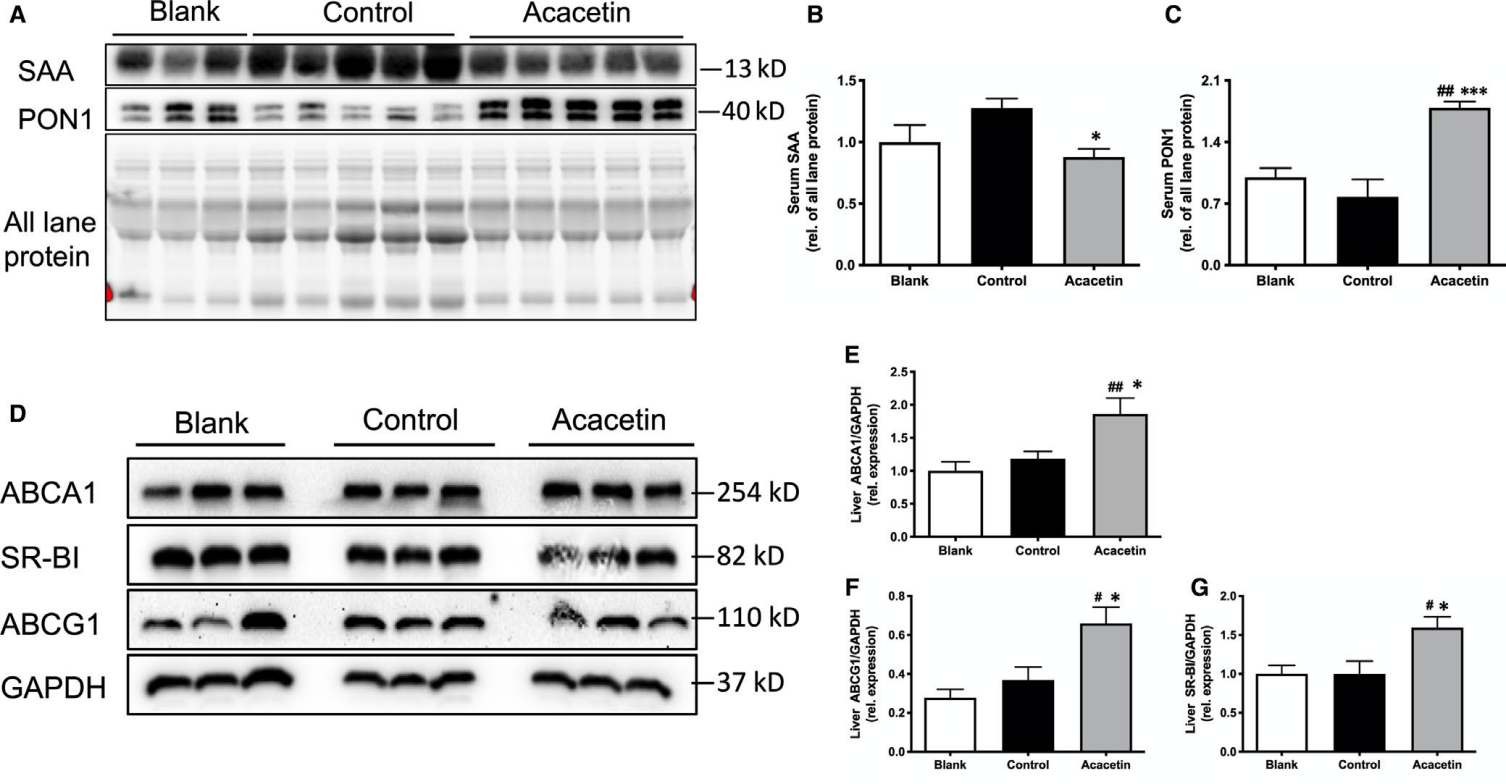
Acacetin ameliorated oxidative and inflammatory stress in the circulation and liver of apoE^−/−^ mice. (A‐C) Plasma SAA and PON1 levels were detected by Western blot. (D‐G) ABCA1, ABCG1, and SR‐BI levels in the liver were determined by Western blot. n = 5‐9 of each group, *P* ^#^ < .05, *P* ^##^ < .01 vs blank group; *P* ^*^ < 0.05, *P* ^***^ < 0.001 vs control group

Anti‐serum amyloid A and PON1 are HDL apolipoproteins: whilst the former has pro‐atherogenic activities,^22^ the latter is instead considered to be atheroprotective and decreases after inflammatory stimuli.^23^, ^24^ We found that the plasma levels of SAA and PON1 were significantly decreased and increased, respectively, in acacetin‐treated mice (Figure 5A‐C). Furthermore, reverse cholesterol transport (RCT) is a process that facilitates cholesterol transport from peripheral organs back to the liver to regulate excessive systemic cholesterol levels. The cholesterol efflux mediated via ABCA1 and ABCG1 outside the cells whilst HDL‐C can be taken up by SR‐BI for degradation of HDL.^25^ We found that ABCA1, SR‐BI and ABCG1 protein levels were up‐regulated in acacetin‐injected mice (Figure 5D,G), which indicates that it may accelerate RCT in the liver. Meanwhile, liver CAT protein expression level was also significantly increased in acacetin‐injected mice relative to that of in controls (Fig. S2A,E). However, Nrf2 and the other oxidoreductases, MsrA, and PON1 expression levels showed no observable differences between the control and acacetin‐treated groups (Fig. S2B,D). These data accordingly suggest that acacetin plays an anti‐oxidative stress role in the liver.

The inflammatory process in the atherosclerotic‐burdened artery may lead to increased blood levels of pro‐inflammatory cytokines, including but not limited to IL‐6 and TNFα.^26^ In this study, plasma samples were diluted to the appropriate ratio and measured using the ELISA method. As shown in Table 1, the concentrations of the pro‐inflammatory factors, IL‐6 and TNFα, were significantly decreased in acacetin‐treated mice compared to that of in controls. However, levels of the anti‐inflammatory factor, IL‐10, were remarkably higher in acacetin‐injected mice, and significantly lower in Western‐fed control mice as opposed to chow‐fed control mice. These results indicated that acacetin improved the anti‐oxidative and anti‐inflammatory status of both the systemic circulation and the liver in Western diet‐fed apoE^−/−^ mice.

## 4 DISCUSSION

Atherosclerosis is no longer thought to be a chronic inflammatory disease characterized solely by dyslipidaemia, but rather by oxidative stress too. ROS‐induced oxidants react with target molecules, including lipids, nucleic acids and proteins, involved in every stage of atherosclerosis, namely during vascular endothelial cell injury, foam cell formation, and smooth muscle cell migration and proliferation.^27^ Intracellular redox status is tightly regulated by oxidant and antioxidant systems, and various studies have demonstrated the importance of anti‐oxidative stress mechanisms in the prevention and treatment of atherosclerosis.^19^, ^28^, ^29^, ^30^

The vascular endothelium, or intima, is the first line of defence between risk factors and atherosclerotic diseases. OxLDL is a key regulator in all pathogenic steps of atherosclerosis: in early stages, oxLDL‐mediated activation of the endothelium induces endothelial dysfunction,^31^ which initiates monocyte recruitment from the circulation, leading to pathological infiltration into the intima and subsequent cellular toxicity. As such, maintaining the balance of endothelium function is particularly important in protection against atherosclerotic plaque development.^32^ Schmerwitz et al found that in HUVEC cells, flavopiridol (a synthetic flavone structure medicine) strongly blocked the expression of endothelial cell adhesion molecules (intercellular adhesion molecule‐1, vascular cell adhesion molecule‐1 and E‐selectin).^33^ Tanigawa et al further discovered that acacetin, from 11 flavones, also significantly inhibited TNFα‐induced E‐selectin expression in HUVECs.^15^ Despite its notable anti‐inflammatory effects demonstrated in endothelial‐related studies, acacetin has also been proven to have anti‐oxidative influences on the ageing of caenorhabdtis elegans.^34^ Moreover, our recent study also demonstrated that acacetin and its pro‐drug confer significant cardioprotection against ischaemia/reperfusion injury via inhibition of oxidative stress, inflammation and apoptosis ex vivo and in vivo.^35^ However, despite the clearly evident potential therapeutic value of acacetin, there is a relative paucity in literature discussing its role in atherosclerosis‐related CVD.

**Figure 6:**
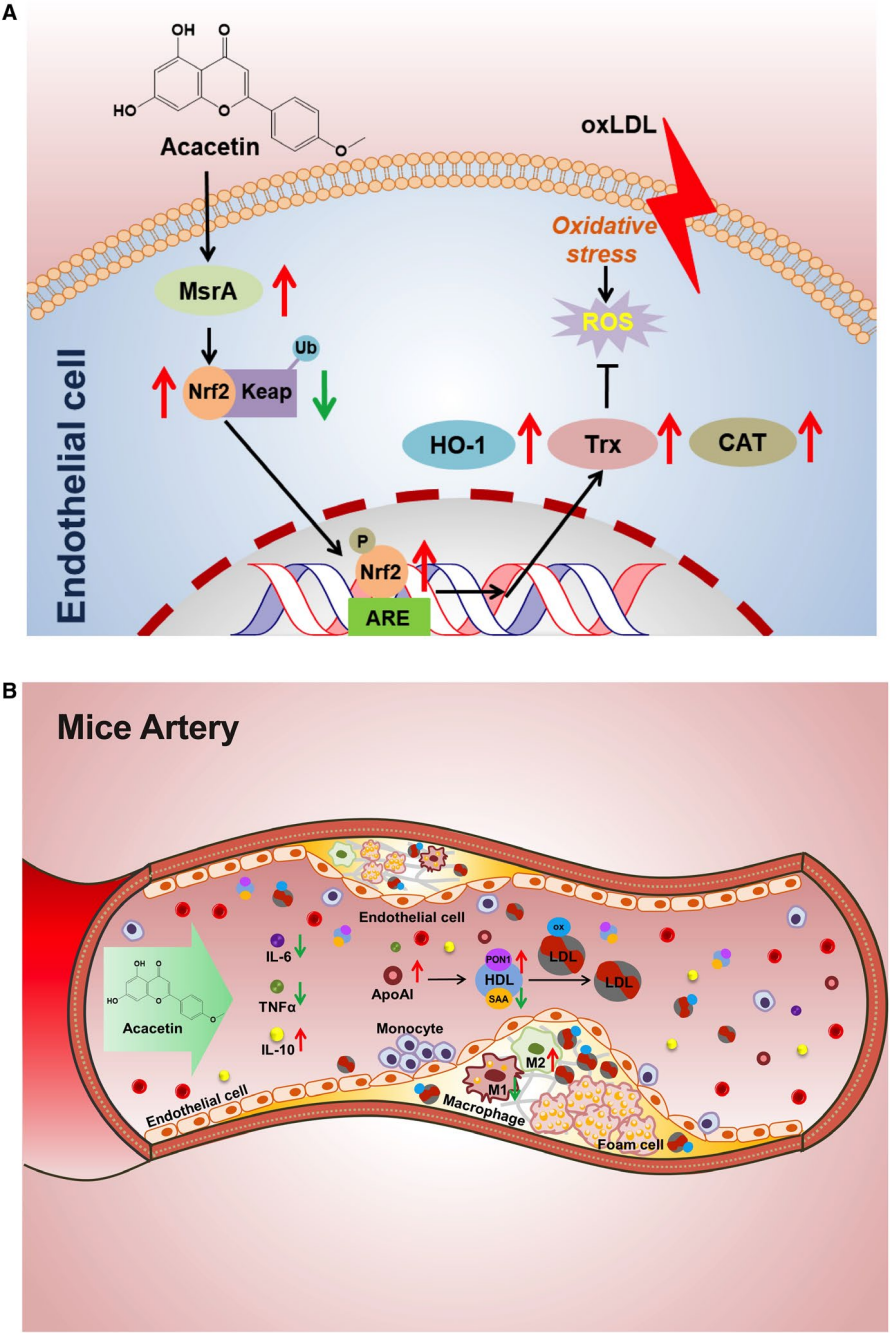
Schematic illustration showing the main protective cascades of molecules of acacetin both in vitro and in vivo. (A) Endothelial cells suffering from oxLDL‐induced oxidative stress and triggers ROS production that reversed by pretreating with acacetin through the MsrA‐Nrf2/Keap1 pathway. (B) Acacetin attenuated atherosclerotic lesions by improving mice circulation anti‐oxidative stress and anti‐inflammation effects

In the present study, we demonstrated that acacetin enhanced EA.hy926 cellular anti‐oxidative effects both on basal levels and under oxidative stress conditions. In vivo, subcutaneous intervention with acacetin twice per day up‐regulated reverse cholesterol transport (RCT)‐related lipoprotein, and anti‐inflammatory factor levels, reduced pro‐inflammatory factor levels, improved mice circulation, and liver anti‐oxidative effects, accelerated liver lipid metabolism, and ultimately attenuated plaque lipid composition (Figure 6).

Acacetin dramatically decreased oxLDL‐induced apoptotic and necrotic cell ratios, potentially through the lowering of intracellular ROS levels. All cells contain a series of antioxidants that can resist and compensate for ROS generation. In our study, acacetin significantly up‐regulated EA.hy926 basal Nrf2 and its downstream HO‐1 protein expression levels. We also found that acacetin increased the production of MsrA, which is one of the notable cellular antioxidant defences involved in many oxidation‐induced diseases.^36^ Under oxidative stress, acacetin confers remarkable anti‐oxidative effects through increasing Nrf2, MsrA, HO‐1 and Trx whilst decreasing Keap1 expression levels. The Nrf2/Keap1 complex is a potent transcriptional activator that plays a central role in the induction of many cytoprotective genes in response to electrophilic and oxidative stress.^37^ Induction of HO‐1 and Trx in a Nrf2‐dependent manner has also been experimentally demonstrated.^38^ Our preliminary results in turn suggest that acacetin enhances EA.hy926 cellular anti‐oxidative effects mainly through MsrA‐related and Nrf2/Keap1 pathways. When Nrf2 protein expression was knocked down by *nrf2* siRNA, the protection provided by acacetin against oxLDL‐induced cellular apoptosis, necrosis and ROS production vanished. Likewise, the protective influence of acacetin similarly disappeared when MsrA expression levels were down‐regulated. This was in stark contrast to when SIRT1 expression was reduced by *sirt1* siRNA, wherein acacetin was still able to exert protection. Moreover, the expression levels of Nrf2, MsrA, Keap1, downstream HO‐1 and Trx did not recover with acacetin under oxLDL stimulation when either the *nrf2* or *msra* gene was silenced.

Further study to determine the relationship between MsrA and the Nrf2/Keap1 pathway was conducted. When the Nrf2 protein was silenced by *nrf2* siRNA, expression levels of the downstream HO‐1 decreased, Keap1 increased and MsrA protein remained unchanged. Surprisingly, we found that both the Nrf2 and downstream HO‐1 expression levels were significantly decreased when MsrA was knocked down by *msra* siRNA (Figure 3I,L). These results differ from that of Pennington et al's findings,^39^ which instead presented an increase in Nrf2, Keap1 and p62 expression in vascular smooth muscle cells from MsrA^−/−^ mice, and in turn proposed that the abundant p62 competes with Nrf2 for binding to Keap1. However, we found an observable increase in Keap1 expression levels when MsrA was down‐regulated in EA.hy926 cells, suggesting that MsrA may be involved in the canonical step of Keap1‐mediated Nrf2 ubiquitination degradation. Such data indicate that MsrA is not only a target of acacetin but also functions upstream of the Nrf2/Keap1 pathway.

However, the means through which acacetin activates Nrf2 is still unknown. After incubation with 3 μmol/L acacetin for 2 hours, we surprisingly found that MsrA and downstream HO‐1 expression levels, as well as the phosphorylated Nrf2^Ser40^ ratio, were all remarkably up‐regulated, whilst the Keap1 expression level was significantly down‐regulated. Such evidence directly suggests that acacetin activates Nrf2 by both phosphorylating the serine residue at the 40th amino acid position and promoting Keap1 degradation. But in this study, we did not find the phosphorylated AMPK^Thr172^ ratio changed.

In the in vivo study, we injected acacetin into Western diet‐fed apoE^‐/‐^ mice to find that such treatment could remarkably reduce the size of atherosclerotic lesions without altering plasma TC and TG levels. As with the cell study, the pNrf2^Ser40^ ratio and MsrA levels were observably increased in acacetin‐treated mice lesions, which may have induced the predominance of the CD206 positive ‘repair’ M2‐polarized macrophage phenotype. Although lipid levels showed no differences between control and acacetin‐injected mice, HDL‐C and apoAI levels were significantly increased in acacetin‐treated mice, which may have enhanced the RCT capacity from peripheral organs. Furthermore, acacetin significantly increased liver ABCA1, SR‐BI, and ABCG1 levels. Whereas SR‐BI former is a cell surface high‐affinity HDL receptor that has a critical role in RCT,^40^ the other functions to mediate cholesterol efflux to HDL or apoAI which is a process essential for HDL formation.^41^ The findings of the present study suggested that acacetin enhanced RCT from peripheral organs, promoted liver‐selective cholesterol uptake by SR‐BI, as well as increased apoAI synthesis and secretion through ABCA1 and ABCG1 without altering plasma lipid levels. In addition to this, injection of acacetin also raised mouse plasma levels of PON1 and IL‐10 and reduced levels of pro‐inflammatory factors, SAA, IL‐6 and TNFα. PON1 is an important anti‐oxidation enzyme that is synthesized in the liver and secreted into the plasma wherein it associates with HDL particles. SAA is likewise also produced in the liver, and its expression is correspondingly increased in response to IL‐6 and TNFα.^24^ However, whilst the expression levels of CAT and SOD2 similarly increased in the liver under pro‐inflammatory factor stimulation, PON1 levels remained unchanged, possibly due to its secretion to the plasma. All these results suggested that acacetin confers an anti‐atherogenic benefit through accelerating lipid metabolism, anti‐oxidation along with anti‐inflammation mechanisms in the circulation and liver.

In our results, both the spleen/body weight ratios and the altered fate of macrophages are changed by acacetin. We speculate that, as the most important immune organ, spleen weight may represent strong immunity with increasing monocytes and enhancing phagocytosis effects (M1 macrophage‐like). YUNG‐LUEN SHIH and colleagues found that bufalin increased the body weight, but reduced liver and spleen weights, and reduced CD3, CD16 and Mac‐3 cell markers. They finally conclude that bufalin may modulate immune responses not only through increasing monocyte (CD11b) population and T‐ and B‐cell proliferation, but also by increasing macrophage phagocytosis in leukaemic mice in vivo.^42^ Marco Busnelli and colleagues found that fenretinide, a synthetic retinoid derivative, could induce spleen abnormally enlarged and markedly increased atherosclerotic lesions at the aortic arch, thoracic and abdominal aorta of fenretinide‐treated mice, just the similar results in our control (western diet) group.^43^ In our present study, we found that mice treated with acacetin had lower spleen/body weight ratio and it may be because acacetin regulates spleen immunity function, decreasing monocytes or promoting monocyte‐macrophage to M2 differentiation.

Interestingly, acacetin‐treated mice had a higher body weight than that of in the control group, a finding in stark contrast to that of Liou et al's, showing that in high fat diet‐fed obese mice, acacetin significantly reduced body weight.^44^ Burke et al recently also reported that citrus flavonoids supplementation to a high fat, cholesterol‐containing diet protected against obesity. These effects may be related to reserve existing obesity, adipocyte size and number through enhanced energy expenditure and increased hepatic fatty acid oxidation.^45^ Potential reasons our differing results include variations in mice type as well as intravenous administrative methods.

## 5 CONCLUSION

In conclusion, our present study demonstrated that the natural flavone acacetin promotes not only a significant reduction in cellular apoptosis through anti‐oxidative stress effects via the MsrA‐Nrf2/Keap1 pathway in vitro, but also halts atherogenesis through accelerating lipid metabolism as well as the anti‐oxidation and anti‐inflammatory capacity of Western diet‐fed apoE^‐/‐^ mice. It may therefore serve as a potential drug candidate for prevention and treatment atherosclerosis‐related CVD.

## CONFLICT OF INTERESTS

The authors have no conflicts of interest to declare.

## AUTHOR CONTRIBUTION

**Yao Wu:** Conceptualization (lead); Investigation (lead); Writing‐original draft (lead). **Fei Song:** Investigation (supporting). **Yunda Li:** Data curation (supporting). **Jing‐Zhou Li:** Investigation (supporting). **Yu‐Kai Cui:** Investigation (equal). **Yi‐Xiang Hong:** Methodology (supporting). **Wei‐Min Han:** Investigation (supporting). **Wei‐Yin Wu:** Resources (supporting). **Ishan Lakhani:** Writing‐review & editing (supporting). **Gang Li:** Writing‐review & editing (lead). **Yan Wang:** Project administration (lead); Resources (lead).

## ETHICS APPROVAL AND CONSENT TO PARTICIPATE

The Xiamen University Ethics Committee approved the protocols according to the Helsinki Declaration.

## Supporting information

Figure S1‐S2

## Data Availability

The data and materials in this study are available on request from the authors.
