# Correction to ‘Acacetin exerts antioxidant potential against atherosclerosis through Nrf2 pathway in apoE ^−/−^ mice’

**DOI:** 10.1111/jcmm.70145

**Published:** 2024-10-28

Wu Y, Song F, Li Y, et al. Acacetin exerts antioxidant potential against atherosclerosis through Nrf2 pathway in apoE^‐/‐^ mice. *J Cell Mol Med*. 2021; **25**: 521–534. doi: 10.1111/jcmm.16106

In the article, the second sentence of the Abstract reads “Acacetin, an anti‐inflammatory and antiarrhythmic, is frequently used in the treatment of myocarditis, albeit its role in managing atherosclerosis is currently unclear.” is correct. This should read “Acacetin is antiarrhythmic and anti‐inflammatory, which is experimentally used in treating myocardial injury.”. The authors confirm all results and conclusions of this article remain unchanged.

We apologize for this error.

