# Supplementary material for: Acacetin exerts antioxidant potential against atherosclerosis through Nrf2 pathway in apoE−/− Mice
Source: J Cell Mol Med. 2020 Nov 26;25(1):521–34. doi: 10.1111/jcmm.16106 (PMC7810944; doi:10.1111/jcmm.16106)
Supplement: Supplementary file 1 — Figure S1‐S2 [file JCMM-25-521-s001.docx]

**Supplemental figures
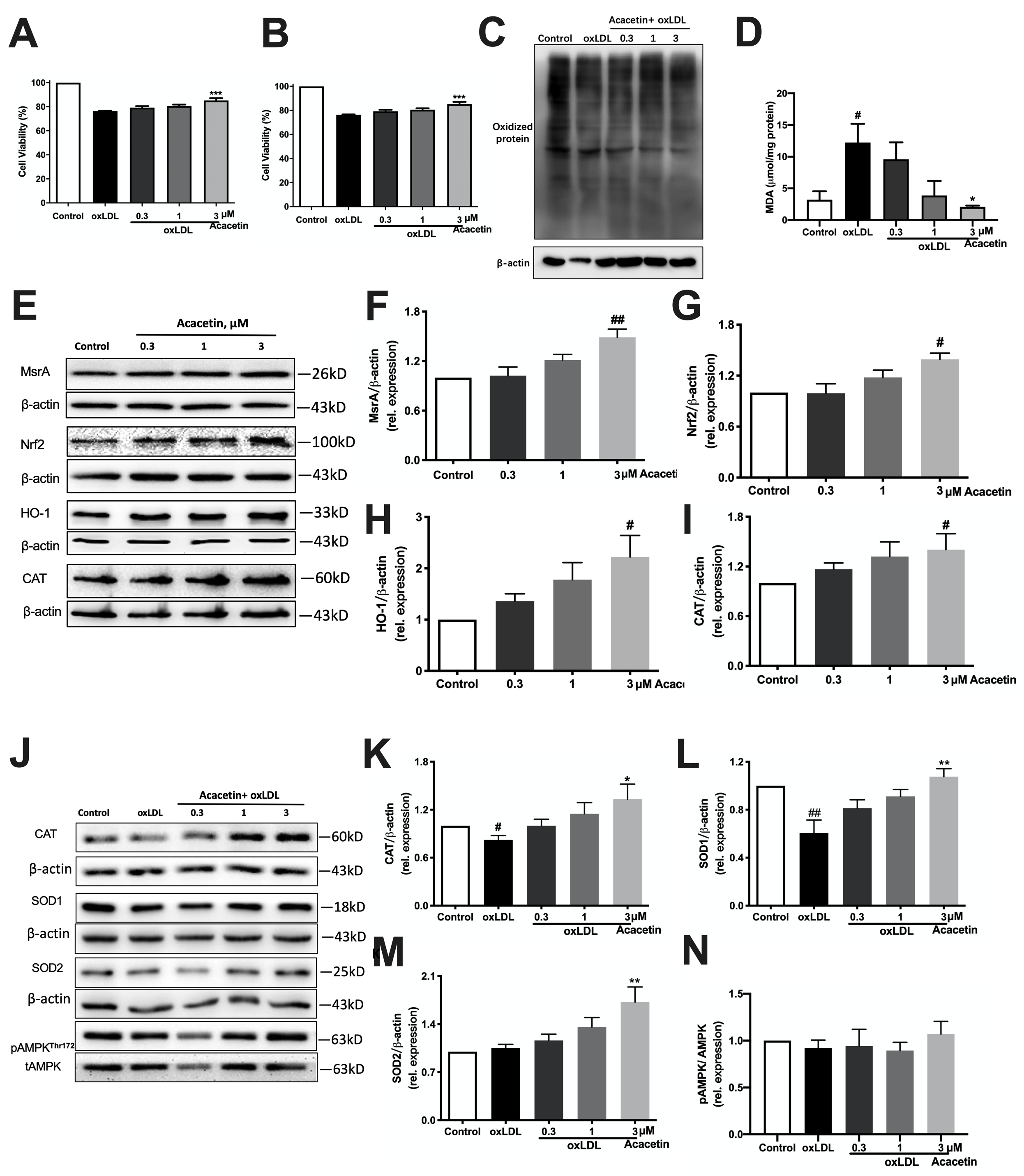
**

**Supplemental figure 1**. Acacetin protected EA.hy926 cell viability by up-regulating cellular anti-oxidative effects under basal and oxidative stress conditions. **(A)** Different concentrations of high oxLDL stimulation of EA.hy926 cells for 20h by the MTT method. **(B)** Cell viability analysis without (control) or with 5μg/mL high oxLDL stimulation in the absence (oxLDL) or presence of 0.3, 1, or 3 μM acacetin were measured by MTT. **(C)** Oxidase protein analysis without (control) or with 5μg/mL high oxLDL stimulation in the absence (oxLDL) or presence of 0.3, 1, or 3 μM acacetin were measured by Oxyblot method. **(D)** MDA production detection without (control) or with 5μg/mL high oxLDL stimulation in the absence (oxLDL) or presence of 0.3, 1, or 3 μM acacetin were measured by MDA detection kit. **(E-I)** MsrA, Nrf2, HO-1 and CAT expression levels were detected under different concentration acacetin (0.3, 1, 3μM) by Western blot. **(J-N)** CAT, SOD1, SOD2, pAMPK^Thr172^ and tAMPK without (control) or with 5μg/mL high oxLDL stimulation in the absence (oxLDL) or presence of 0.3, 1, or 3 μM acacetin were measured by Western blot. n=5 of each group, *p*^#^<0.05, *p*^##^<0.01 vs. control group; *p*^*^<0.05, *p*^**^<0.01 vs. oxLDL group.

**
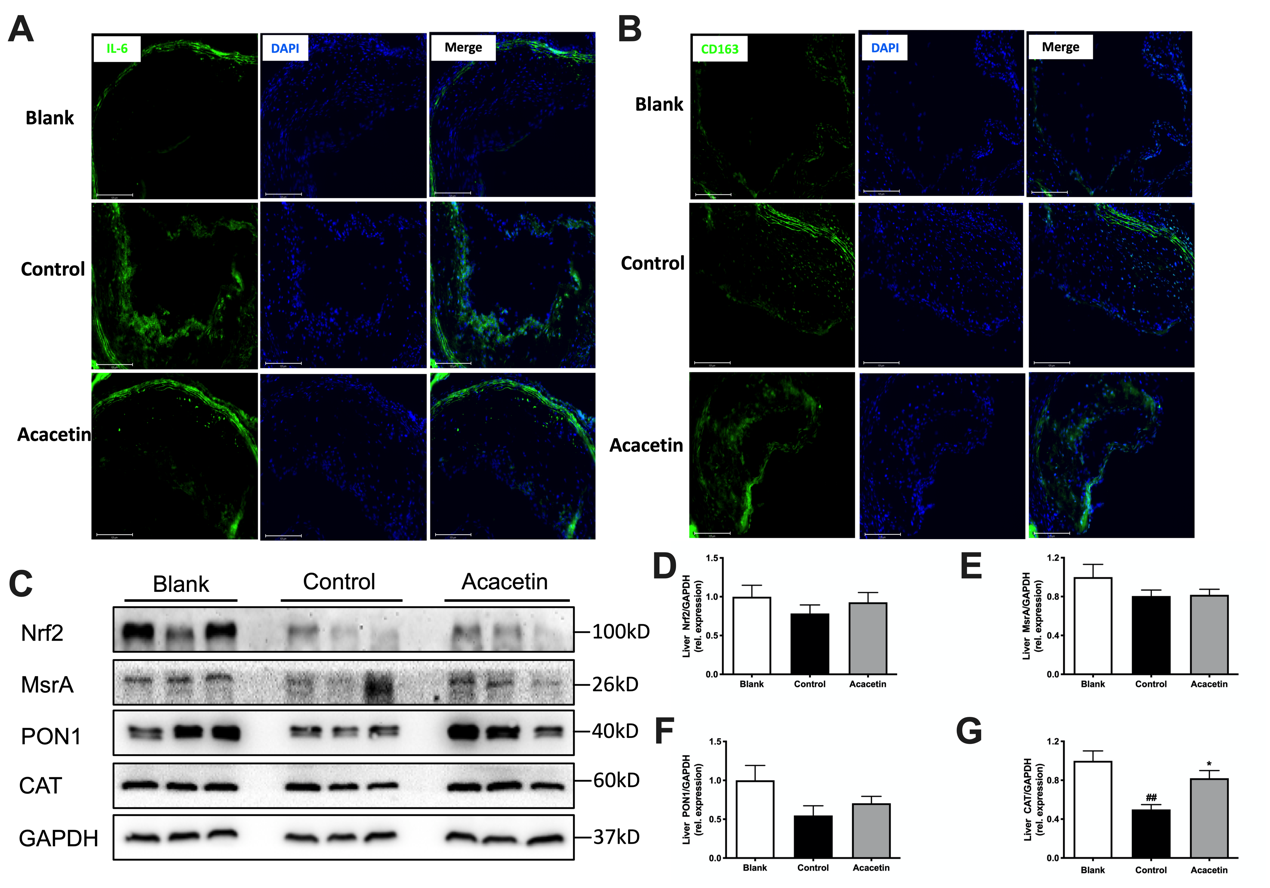
**

**Supplemental figure 2**. Acacetin up-regulated anti-oxidation in the liver of apoE^-/-^ mice. **(A-B)** The M1 (positive with IL-6) and M2 (Positive with CD163) macrophages profile in aortas were detected by immunofluorescence assay. **(C-G)** Liver Nrf2, MsrA, PON1 and CAT were measured by Western blot. n=5-9 of each group, *p*^##^<0.01 vs. blank group; *p*^*^<0.05 vs. control group.
